# Label-free detection of γ-aminobutyric acid based on silicon nanowire biosensor

**DOI:** 10.1186/s40580-019-0184-3

**Published:** 2019-05-01

**Authors:** Jin-Ho Lee, Eun-Ji Chae, Soo-jeong Park, Jeong-Woo Choi

**Affiliations:** 1 Department of Chemical and Biomolecular Engineering, Sogang University, Seoul, 04107 Korea; 2 Department of Chemistry and Chemical Biology, Rutgers, The State University of New Jersey, Piscataway, NJ 08854 USA; 3 Research Center for Disease Biophysics of Sogang-Harvard, Sogang University, Seoul, 04107 Korea

**Keywords:** γ-Aminobutyric acid (GABA), Neurotransmitter, Silicon nanowire field-effect device, Immunosensor, Biochip

## Abstract

γ-Aminobutyric acid (GABA) is an important inhibitory neurotransmitter in the central nervous system (CNS), which acts as a major biomarker for neurological disorders such as Parkinson’s disease and Meningitis. To this end, the precise measurement of GABA molecule arisen as an important subject for the effective diagnosis and treatment of neurological disorders. However, yet highly sensitive biosensor systems which can analyze a wide range of GABA molecule in a fast response manner have not been reported. In this study, for the first time, a silicon nanowire field-effect transistor (FET) device based immunosensor was developed to detect GABA molecule. Zig-zag shaped silicon nanowires has been fabricated by electron beam lithography and the electrical property p-type FET device was validated through semiconductor analyzer. The optimal immobilizing condition of antibody against GABA molecule was determined by the fluorescent signal measurement. Various concentrations of GABA ranging from 970 fM to 9.7 μM were sensitively measured by conductance change on silicon nanowire-based through the immunoreactions. Further, owing to the ease of miniaturization and label-free system, we believe that the suggested device system has a potential to be utilized for an implantable biosensor to detect neurotransmitter in the brain and can create new opportunities in the field of diagnosis and treatment of neurological disorders.

## Introduction

It is estimated that the tendency of patients suffering from neurological disorder is increasing every year. However, the difficulty in diagnosing the neurological disorders hampers the treatment efficiency [1, 2]. For example, one of the most common neurological disorders such as Parkinson’s disease and Meningitis are distinguished by PET, MRI, and SPECT without practical and objective instrument [3–5]. Although, bacteria culturing is considered as one of a precise method for diagnosing Meningitis; however, it is time-consuming and appropriate to only bacterial-induced Meningitis [6]. As well, Parkinson’s disease can be only precisely diagnosed by examining the brain after the patient’s death [7]. Thus, the urgency to develop an effective and simple diagnosing technique of neurological disorder is clear.

There are many causes for triggering neurological disorder, but one of the major cause of the neurological disorder is due to the imbalance of excitatory neurotransmitters such as GABA (γ-aminobutyric acid) [8, 9]. For example, for the patients who are suffering Meningitis has significantly lower GABA concentration in CSF (range from 0.11 to 0.15 nM) compare to normal people (range from 167 to 249 nM). And for the patients who are suffering Parkinson’s disease has significantly higher GABA concentration in CSF (range from 0.7 to 1.5 μM), which indicates the potential use of GABA as a good biomarker for neurological disorders [10, 11]. Recently, numerous detection methods have been developed to monitor GABA, but still, several problems exist. For instance, due to the similarity in a molecular structure with other macromolecules, it is difficult to detect GABA accurately from overlapping signals [12]. Comparably, an optical and piezoelectric method is utilized to develop immuno-sensor for high specificity, however, the detection range and limit are not appropriate enough to diagnose neurological disorders precisely [13–16]. Also, there are other instruments which are more sensitive than piezoelectric immuno-sensor such as liquid chromatography, but they are inefficient and time-consuming because of sample pretreatments [17]. Hence, another alternative detection system must be needed to overcome these problems of the conventional GABA detection systems.

**Fig.1:**
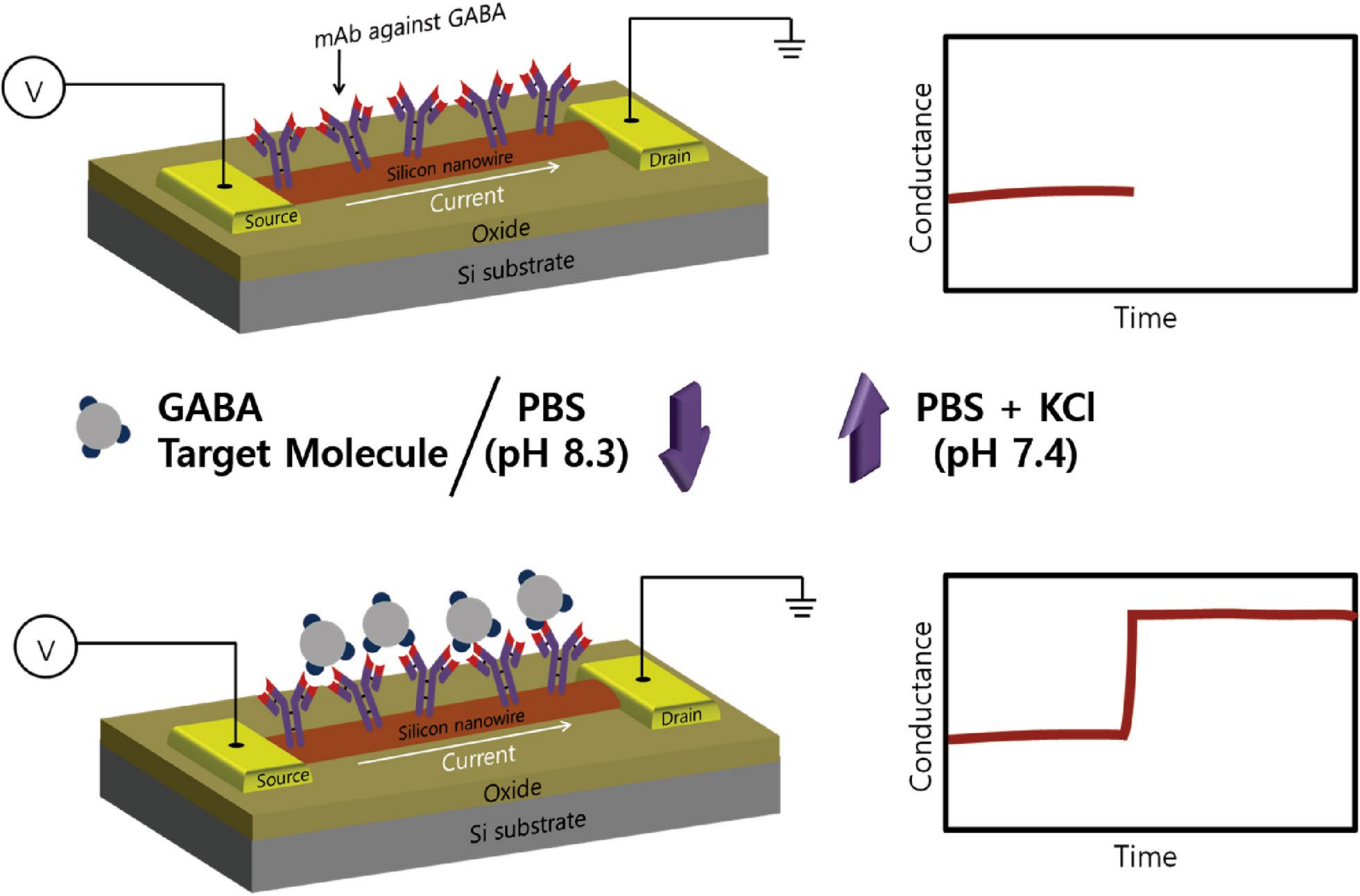
Schematic diagram of the silicon nanowire-based on field effect transistor device for GABA detection. When GABA was applied to this system, the conductance is increase due to the immunoreaction

Recently, a semiconducting nanowire-based field-effect transistor (FET)-based biosensors have gained much interest owing to its outstanding properties and efficiency. As sensing elements, the FET device serves to overcome many obstacles faced by current sensing technologies. For example, significant advantages such as portability, high sensitivity, fast response, low manufacturing cost, and label-free detection procedure have provided a clear aspect to develop FET-based biosensors for analyzing bio/chemical molecules. To this end, in this study silicon nanowire, FET device was utilized for the first time to develop a highly sensitive biosensor for detecting GABA molecule in a fast response manner (Fig. 1). Different concentrations of GABA molecule (range from 970 fM to 9.7 μM) in a wide range were sensitively analyzed by conductance change based on the immunoreactions.

## Method/experimental

### Materials

Monoclonal antibody against GABA and FITC (Fluorescein isothiocyanate) conjugated antibody were purchased from Abcam (Cambridge, USA). γ-Aminobutyric acid (GABA) (C_4_H_9_NO_2_) was purchased from Sigma-Aldrich (Cambridge, UK). P-type silicon nanowire was fabricated on SOI (Silicon on Insulator) wafers from Nanopep Center (Daejeon, Korea). Distilled and deionized Millipore [(Milli-Q) water (DDW > 18 MΩ)] and nitrogen gas were used for cleaning and drying. Tri-ethoxy-silyl-butyl-aldehyde (TESBA) (C_10_H_22_O_4_Si) was purchased from Gelest, Inc. (USA). Sulfuric acid, hydrogen peroxide, and all other chemicals were purchased from the Aldrich Chemical Co. (USA) and used without further purification.

### Fabrication of silicon nanowires

A 4-in. silicon on insulator (SOI) wafer which initially had a 100 nm silicon layer on a 375 nm buffered-oxide insulating was used as a starting material. The top silicon layer was doped as p-type by introducing approximately 10^14^–10^15^/cm^3^ Boron and its resistivity was measured to be 10–20 Ω/cm^2^. By using electron beam lithography, 100 nm zig-zag shaped p-type silicon nanowire was fabricated on SOI wafer and followed by 150 nm chrome and 30 nm gold deposition to make source and drain electrode after etching process using CF_4_ gas. The structure of fabricated silicon nanowire on SOI wafer was verified with scanning electron microscopy (SEM) (JSM-6700, 15–30 kV).

### Immobilization of antibody on silicon nanowire by self assembly

Fabricated silicon nanowire was treated with O_2_ plasma at 50 W and 50 sccm O_2_ for 60 s to attach hydroxyl group [18]. After treating with O_2_ plasma, the optimized concentration of antibody (50 μg/ml) was immobilized by TESBA solution and utilized for the detection of GABA.

### Electrical detection of GABA

B1500A semiconductor parameter analyzer (Agilent, Inc, USA) was used to measure the conductivity changes on the silicon nanowire with negatively charged GABA solution. When conductance of the silicon nanowire patterned chip was stabilized at 1 V on the gate electrode (V_G_) and 0.5 V on the source to drain electrode (V_SD_), GABA solution was applied to the system. After the change of conductance became a stable state, 3 M KCl solution was applied to dissociate the antibody-antigen interaction and 10 mM phosphate-buffered saline (PBS) buffer was applied repeatedly until the conductance decreased at the baseline [19]. Then, various concentration of GABA solution (range from 970 fM to 9.7 μM) was applied to the system for the analysis.

## Results and discussion

### Generation of silicon nanowire

**Fig. 2:**
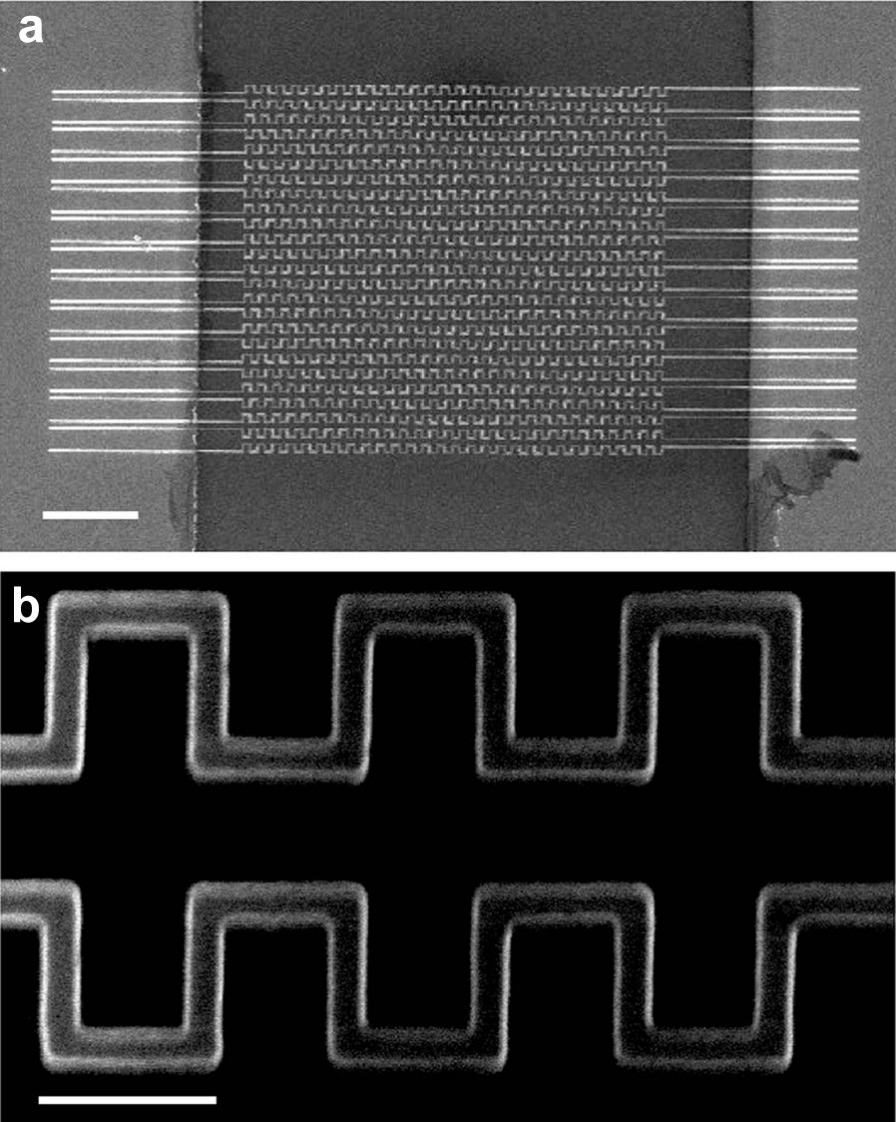
**a**,** b** Scanning electron microscopy image of the fabricated nanowire pattern on SOI wafer. (Scale bar **a** 5 μm; **b** 500 nm)

Zig-zag shaped 100 nm wide silicon nanowire was fabricated on SOI wafer by electron-beam lithography. The structure of fabricated silicon nanowire pattern on SOI wafer was verified by SEM images (Fig. 2a, b). 25 silicon nanowires were fabricated in parallel between source and drain electrodes. The structure of fabricated silicon nanowire was carefully designed to have a zig-zag shape to have more active surface areas than that of single straight wire. Each silicon nanowire was fabricated at a distance from 250 to 900 nm to avoid interruption between each wire.

**Fig. 3:**
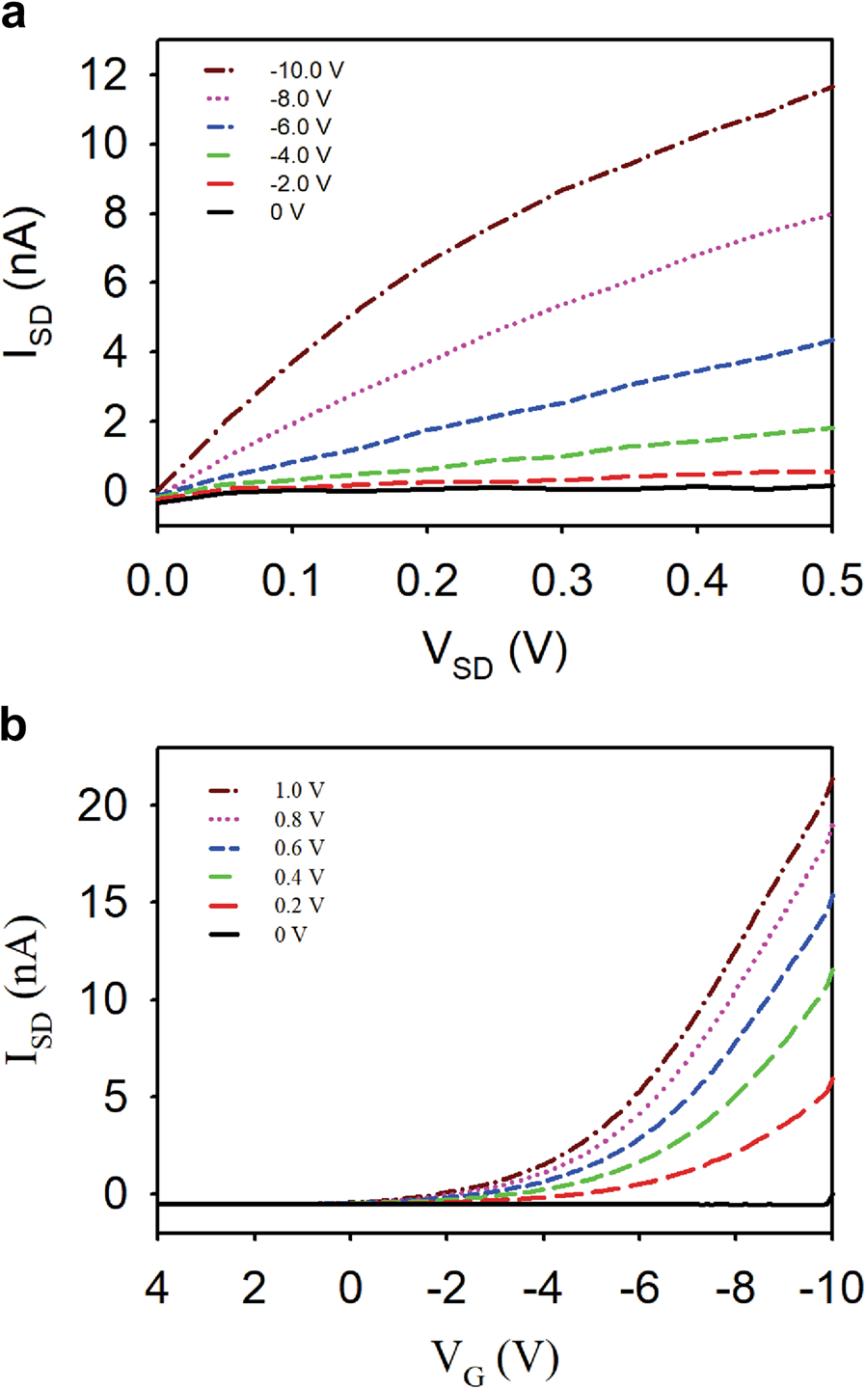
Electrical properties of silicon nanowire-based FET device. **a** Source–drain current (I_SD_) versus source–drain voltage (V_SD_) plots at different gate voltages. **b** Source–drain current (I_SD_) versus gate voltage (V_G_) at a different source–drain voltage (V_SD_) of the fabricated nanowire pattern on SOI wafer

In general FET devices, the current flows through the electrical channel between two metal source and drain electrodes. The role of the gate electrode is to increase or decrease the current channel between the source and drain [19, 20]. In case of the p-type silicon nanowire, which is used in this experiment, the conductance will increase as applying negative gate voltage, or decrease as applying positive gate voltage because electron holes work as a carrier in the p-type silicon nanowire [19]. The electrical property of fabricated zig-zag shaped silicon nanowire on SOI wafer was validated by semiconductor parameter analyzer (Fig. 3a, b). As shown Fig. 3a, the source−drain current increased when the source-drain voltage was increased within different gate voltages (range from 0 to − 10 V). Comparably, it is figured out that source–drain current also increased as the higher negative potential is given to the gate voltage. The I_SD_–V_G_ measurements show that the increase in current between the source (V_SD_) and drain (I_SD_) for below − 2 V on V_G_ was believed to be due to gate leakage and the I_SD_ was decreased as the V_SD_ was decreased (Fig. 3b). These data clearly demonstrate the properties of p-type silicon nanowire.

### Optimization of antibody immobilization on the silicon surface

**Fig. 4:**
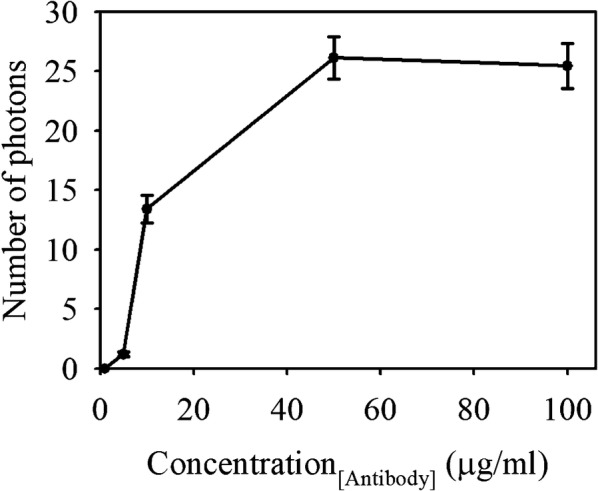
Optimization of antibody immobilization on the silicon surface by measuring the number of photons through the spectrometer

To maximize the sensitivity through the immunoreaction, the optimal condition for antibody immobilization on silicon nanowire was investigated by using FITC conjugated IgG antibody. 2 × 2 cm^2^ sized silicon substrate was pre-treated with O_2_ plasma at 50 W and 50 sccm O_2_ for 60 s to uniformly form a hydroxyl group on the silicon substrate. Silicon substrate was immersed in TESBA solution composed of 2% v/v TESBA in ethyl alcohol anhydrous/DDW (95%/5%) for 1 h at room temperature to form an aldehyde-terminated functional group on the surface of the silicon substrate by self-assembled monolayers technique [21]. After sequential washing with ethanol and DDW, the substrate was incubated in the different concentrated solutions of FITC conjugated IgG antibody (range from 1 to 100 μg/ml) for 4 h under 4 °C. In addition, 1 M of ethanolamine (C_2_H_7_NO) was applied to silicon substrate for about 1 h to block uncoupled functional aldehydes groups. After washing silicon nanowire with PBS (pH 7.4), the number of photons per unit area was measured by using N-spectra (NT-MDT, Russia). Since it is well known that FITC has an absorption maximum at 494 nm and an emission maximum of 521 nm, the spectra were obtained using laser-emitting light at a 488 nm wavelength with an irradiation laser power of 3 mW on the sample plane. In addition, the emission range was arranged from 490 to 620 nm because the broad emission peak was appeared to locate at this range. Total (32 × 32 per 100 μm × 100 μm) points were scanned for 5 s each and the mean value of the number of photons was analyzed. Examining the number of photons through light scattering from the antibodies conjugated with FITC, the optimal condition of the antibody immobilization was able to predict. As shown in Fig. 4, the number of photons was increased continuously up to 50 μg/ml and saturated above. Thus, 50 μg/ml was selected as the optimal concentration of immobilizing antibody on a silicon substrate and used for further experiments.

### Detection of GABA molecule through silicon nanowire-based FET device

**Fig. 5:**
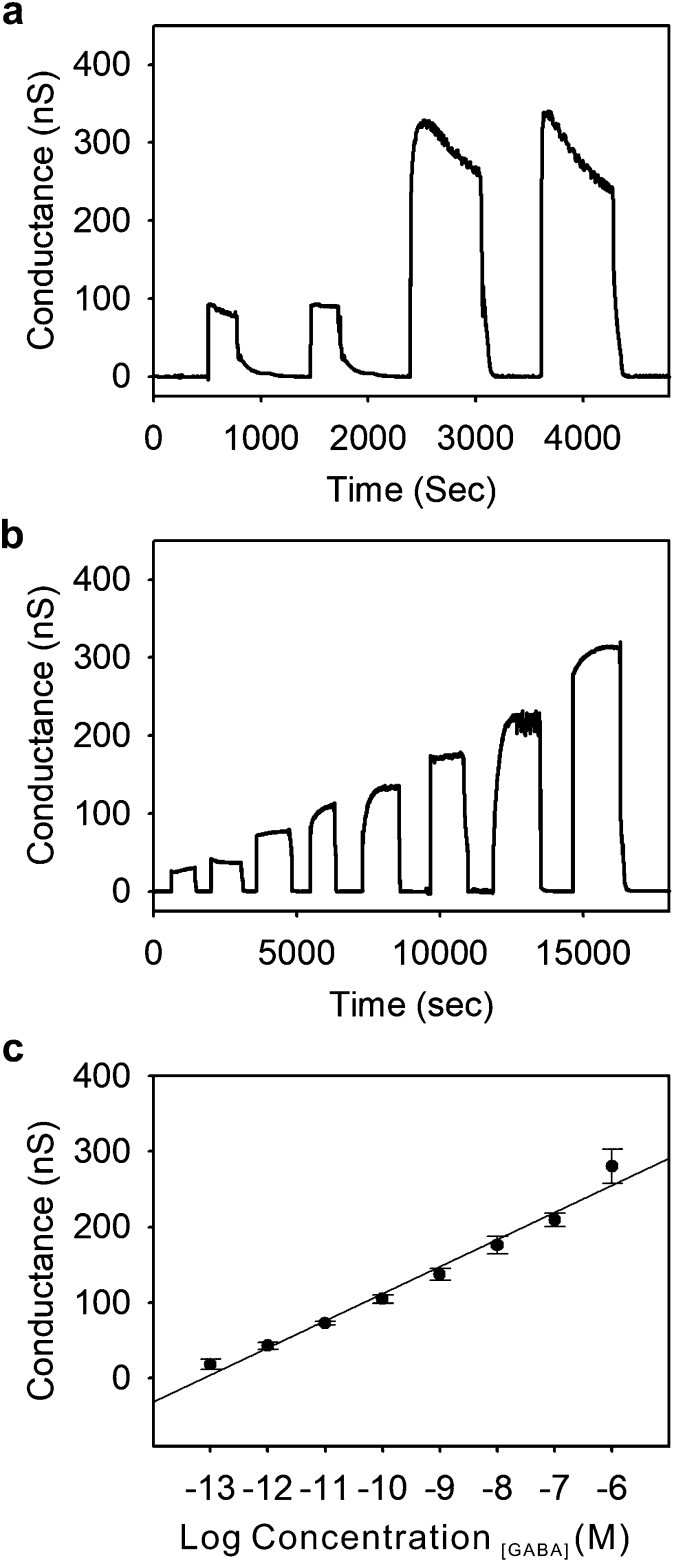
Monitoring GABA molecule through silicon nanowire-based FET device. **a** Reproducible conductance signal observed through selected concentrations of GABA molecule. **b** Conductance versus time data on GABA antibody modified p-type silicon nanowire after applying the target molecule (GABA) after applying the target molecule (GABA) range from 970 fM to 9.7 μM. **c** The calculated linear correlations between the concentration of GABA and conductance change of a p-type silicon nanowire-based FET device

In a standard FET, gate voltage performs a very important role in controlling the current between the source and a drain electrode which is connected to silicon on the silicon oxide substrate. Particularly, in a p-type semiconducting silicon nanowire, the binding of negative charged molecules to the surface (which works similar to applying a negative gate voltage) will lead to the accumulation of charge carriers (holes) and subsequently increase in conductance due to channel opening in the semiconducting silicon nanowire. In an opposite manner, the binding of positively charged molecules (which works similar to applying a positive gate voltage) will deplete holes and reduce the conductance [22]. As the net charge of GABA is considered to be negative in PBS (pH 8.4) buffer solution based on the pI value (pI ~ 7.3) [23]. Upon the immunoreaction between negative charged GABA molecules and antibodies attached on p-type silicon nanowire, will be analogous enough to accumulate charge carriers in a silicon nanowire and leads to the remarkable increase in a conductance (Fig. 1). Moreover, 3 M KCl solutions were used to dissociate antibody-antigen binding interactions effectively without permanently degrading the antibody protein structure to prove the reusability. As it can be seen in Fig. 5a, the conductance was changed after GABA solution was applied, and returned to the baseline subsequently as 3 M KCl solution dissociated negatively charged GABA from immunoreactions. Moreover, the reproducible signal was able to be obtained with the same concentration of applied GABA molecules for both low (97 pM) and high (9.7 μM) concentrations (Fig. 5a). As applying the different concentrations of negatively charged GABA solutions (pH 8.4) from 970 fM to 9.7 μM to silicon nanowire-based, FET device, the current flowing between the source and drain electrode was increased, respectively (Fig. 5b). As shown in Fig. 5c, the conductance change was nearly proportional to the different concentration of GABA solutions with R^2^ = 0.979. In conclusion, the developed system was able to detect different concentrations of GABA solutions in a wide range in label-free with high accuracy, which fully covers the diagnosis range of Parkinson’s disease and Meningitis with high sensitivity and fast response time.

## Conclusion

In this study, silicon nanowire-based FET device was fabricated to detect GABA as a biomarker of neurological disorders such as Parkinson’s disease and Meningitis for the first time. The optimal immobilizing concentration of antibody was determined to be 50 μg/ml by the fluorescent method and the electrical property of silicon nanowire was clearly verified to p-type. Variant concentrations (from 970 fM to 9.7 μM) of GABA were successfully measured on silicon nanowire field-effect device based on the immunoreactions. The conductance change was proportional to the concentration of GABA molecule accurately in a wide range, demonstrating that this system could work as an important index of diagnosing neurological disorders. In addition, since this FET based system is easy to be miniaturized and does not need any labeling materials, we believe that the present system could be utilized to develop the implantable biochip to detect neurotransmitter in the brain as well.
